# Exploring the Unique Professional Identity of Hospital Teachers

**DOI:** 10.5334/cie.46

**Published:** 2022-11-02

**Authors:** Meirav Hen, Maskit Gilan-Shochat

**Affiliations:** 1 Tel-Hai academic college, IL; 2 Schneider Children’s’ medical center, IL

**Keywords:** Professional identity, Hospital teachers, Hospital school, Teaching context

## Abstract

Teachers’ professional identity is a key factor in their motivation, effectiveness, and job satisfaction. The present study examined hospital teachers perceived professional identity based on their work experience in a unique educational environment. Thirty-seven hospital teachers reflected on their professional identity and other personal and professional aspects of their work experience in semi-structured interviews. Hospital teachers reported a multilayer professional identity and described their work environment and teaching as complex, different from teaching in the regular school, and satisfying. Scientifically clarifying their unique professional identity is important for the development of this unique profession and for enhancing their professional confidence and well-being.

Professional identity is considered a key factor in teachers’ motivation, effectiveness and retention, job satisfaction, and commitment (Canrinus et al., 2011). It is believed that teacher professional identity is continually being formed and reformed through their internalizing the external environment, negotiating interactions, and externalizing themselves to others (Rodgers & Scott, 2008). During their careers teachers construct and reconstruct a conceptual sense of who they are, and this is manifested through what they do (Cohen, 2010).

For teachers whose students are hospitalized children and whose school setting is a medical center, the teaching context and role change constantly and dramatically (Benigno & Fante, 2020), likely affecting their sense of professional identity in many ways. The changing landscape of children with different physical, emotional, and cognitive needs, the inability to predict who will be your students the next day and how long a particular student will be under your tutelage, in addition to working with a multi-professional staff, are some of the challenges that characterize hospital teachers’ professional milieu (Äärelä, Määttä, & Uusiautti, 2016, 2018; Csinády, 2015). Although teachers have been working in hospitals for many years, the topic of this unique professional identity has not been addressed in the professional literature (Andreatta, et al., 2015; Hopkins, 2016).

This study examined Israeli hospital teachers’ perceptions of their professional identity based on their perceived work context, teaching practice and their overall professional lives. The aim of this study is to contribute to the growing literature on hospital teachers, and to help advance and consolidate this unique profession.

## Teachers’ professional identity

Teachers’ professional identity generally pertains to how teachers view themselves based on their own interpretations of their continuing interaction with their context (Rodgers & Scott, 2008). This is a dynamic, ongoing process which entails making sense of and reinterpreting one’s own values and experiences that may be influenced by personal, social, and cognitive factors (Henry, 2016). Teachers’ professional identity plays a key role in decisions teachers make about their teaching practices, the content they teach, the kind of relationships they maintain with their students, where they place their efforts, as well as, whether and how they seek out professional development opportunities (Pillen, den Brok & Beijaard, 2013). The formation of teachers’ professional identity has been studied mostly through a social theoretical framework investigating the contribution of four broad factors: (1) reflective activities, (2) learning communities, (3) context, and (4) prior experiences (Izadinia, 2013; 2015). These studies concluded that teachers’ professional identity is a complex and dynamic concept strongly influenced by their accumulated knowledge during training and their interpretation of the experienced working context and practice (Henry, 2016).

Akkerman & Meijer (2011) discussed teachers’ professional identity in the theoretical framework of the dialogical approach. They suggested that in the postmodern view identity is no longer a unified framework, but rather reflects the multiple social worlds in which people engage. Thus, they speculated that teacher professional identity should be studied from the individual’s wide perspective, blending from within the self, unitary and multiple, continuous and discontinuous also, individual and social. They argued that the teacher experiences an ongoing process of negotiating and inter-relating multiple positions of the self (i.e., I-positions) in such a way that a coherent and consistent sense of self is maintained throughout various participations and self-investments in one’s working life (Akkerman & Meijer, 2011).

Rodgers and Scott (2008) claimed that teachers should work towards an awareness of their identity and the contexts, relationships, and emotions that shape them, claiming the authority of their own voice. They concluded that teachers’ identity is dependent upon the contexts in which teachers immerse themselves and the ways in which they do so (Rodgers & Scott, 2008). Flores and Day (2006), argue that teachers balance three relevant dimensions in their work: a personal dimension (teachers’ life outside the school), a professional dimension (social and policy expectations of what a good teacher is, and teachers’ own educational ideals) and a situational dimension (the direct working environment of the teacher). Through the interaction of these dimensions, different professional identities are formed (Flores & Day, 2006). Along the same lines Beijaard et al. (2004) emphasized that the context of the teaching – what surrounds the teacher, what other people expect from him/her, and how the teacher allows all this to impact them (the teaching landscape) – seems to be a very crucial factor in the formation of teachers’ identity.

Based on the above literature of the formation of teacher’s professional identity (Flores & Day, 2006; Henry, 2016; Rodgers & Scott, 2008), and based on the assumption that hospital schools are unique and include multiple teaching landscapes (Benigno & Fante, 2020; Hopkins, 2016), hospital teachers’ personal and professional experiences may integrate into a very different experience of teachers’ professional identity. In the following section the unique context of hospital schools and some of the roles in which hospital teachers engage will be presented.

## Hospital Teachers

Some children, by virtue of acute or chronic medical problems, are unable to attend school on a regular basis (LeHo Project, 2015). They are either at home or in a hospital and their educational needs are met in out-of-school circumstances (Capurso & Dennis, 2017). To support these children’s educational rights, hospital schools were established in many hospitals around the globe (Äärelä, et al., 2016; Benigno & Fante, 2020). In most countries teaching in hospital schools is carried out by qualified teachers (Hen, 2020; Benigno & Fante, 2020; Csinády, 2015; Maor & Mitchem, 2018) and is based on the curriculum of the mainstream schools (Hopkins, 2016; Steinke et al., 2016).

However, the educational milieu, the teaching tasks and the student population present a unique setting that differs tremendously from what teachers are used to in the base school system (Capurso & Dennis, 2017; Csinady, 2015). Most hospital teachers do not work in a set schedule. Every morning they meet the hospitalized children who were admitted to the ward, and personal lessons are conducted according to their educational and emotional needs (Äärelä, et al., 2016). Hospital teachers teach the base school curriculum adjusted to the student’s age and knowledge (Kapelaki et al., 2003), but they also teach unique classes relating to a medical condition or a procedure (Steinke et al., 2016), help the hospitalized student process hospital- or illness-related experiences through expressive means (Regev et al., 2015), and act as an intermediary between the child and the medical and nursing staff (Eaton, 2012). In addition, hospital teachers often contact and sustain a connection between the child and his/her school in the community (Maor & Mitchem, 2018), and prepare students for reentry to their enrolled school after long term hospitalization (Hen, 2022; Choquette, et al, 2016).

Hospital teachers often work at the child’s bedside, considering the physical and emotional state of the student, competing with the demands of health professionals, visitors, families, and assorted distractions (Andreatta, et al., 2015; Benigno et al., 2020). Therefore, teachers must be psychologically oriented, have good social -emotional skills (Hen, 2020), and use their ability to observe and be intuitive to employ the proper methods and words to motivate the hospitalized child (Hopkins, 2016). Hospital teachers are required to have a high degree of flexibility, dynamism, initiative, and creativity to be able to intensively adapt to an ever-changing work environment (Csinady, 2015; Steinke et al., 2016). In addition, it should be well thought-out that hospitalized children are usually suffering from pain, fear, helplessness, uncertainty, loss of routine and loss of independence, all of which must be taken into consideration and addressed by the hospital teacher (Äärelä, et al, 2016, 2018).

In most cases, these special-education, or specific-subject teachers do not receive specific training for working with inpatient children, so that they report a constant need to educate themselves in alternative ways of teaching (Beningo et al., 2018; Maor & Mitchem, 2018). The hospital teacher’s educational endeavors may not be considered lifesaving, but the literature indicates that they significantly contribute to the quality of life and conduct of the hospitalized child, his/her collaboration with the medical staff, and his/her personal internalization of the hospitalization experience (Capurso & Dennis, 2017). A child who meets with a teacher in the hospital realizes that even in this strange and unexpected situation, there is a familiar figure who can help him cope with his/her current uncomfortable and frightening experience by immersing him into a recognizable context, and subsequently return him to his healthy world and stronger self (Goodman, 1988).

Finally, in addition to the educational tasks, hospital teachers cooperate with the medical and allied-health professionals in the hospital. At most times medical and some paramedical decisions naturally take priority over educational matters and the educational teams cooperate and support the work of the medical team (Csinady, 2015; Steinke et al., 2016).

## The Present Study

The above literature emphasizes the unique and complex teaching context and roles of hospital teachers, as well as, with the significant impact of teachers’ professional identity in their practice and professional lives. Following the literature on teachers’ professional identity (Akkerman & Meijer, 2011; Rodgers & Scott, 2008) the present study used a qualitative research method based on semi-structured interviews to examine how hospital teachers perceive and negotiate their professional identity as reflected in their own voices, by their personal and professional experiences.

## Methods

### Participants

Data were collected from 37 hospital teachers who were approached by the researchers and volunteered to participate in this study. All participants are part of the educational staff in three children’s medical centers in our country’s central region, receiving over 250,000 visits from patients annually. These medical centers also serve as an international referral center for many complicated procedures in the Middle East and Europe. The study was approved by the largest medical center’s ethics committee in July 2018 (# 142-2018-A) and approved by the two other centers accordingly.

All hospital teachers participating in this study were female (most hospital teachers in our country are females), between the ages 27–52 with an average teaching experience of 10 years in general, and six years in a hospital school. All participants are certified teachers, 20 holding a B.Ed. degree, six with a B.S in sciences and 11 with an M.Ed. degree. Ten of the participating teachers were certified special education teachers; 34 were Jewish, two Moslem and one Christian.

### Data collection

Hospital teachers signed a consent form and were invited to be individually interviewed in a private room in the medical center where they are employed. Upon agreement, a trained research assistant interviewed them using a semi-structured interview protocol developed by the researchers and based on the concept detailed above of teachers’ professional identity (Akkerman & Meijer, 2011; Rodgers & Scott, 2008). All interviews were conducted by six trained research assistants (Psychology students) in the medical centers. Hospital teachers were requested to reflect on different aspects of their professional self (including their personal feelings, views, expectations, experiences, and relationships at work, to reflect on their professional teaching environment (context) and roles and how this influences their unique professional identity. For example, teachers were asked about their professional goals and expectations, their actual day-to-day experiences at work, their relationship with hospitalized children and parents, the medical staff, the educational staff, and the way in which they explain their professional role to others.

### Data analysis

The main goal of this qualitative analysis was to identify categories that would allow us to better understand hospital teachers’ professional identity. We employed the consensual qualitative research (CQR) method (Hill et al., 2005) to capture the unique experiences of hospital teachers and make contributions that may not be available in larger quantitative inquiries. All analysis decisions were made by consensus (Hill et al. 2005).

In the initial step of data analysis all interviews were transcribed by the research team that included four BA psychology students, two colleagues who are well-acquainted with the teachers’ professional identity literature, and the main researcher. To ensure accuracy, the research team switched transcripts and checked them against the recordings. The next step was to develop an initial list of domains based on the interview protocol (Hill et al., 2005). The research team members then independently assigned each aspect of the transcripts to these domains while remaining alert for other domains that were not included in the initial list. After the transcripts were coded into domains, the team members discussed and assigned core ideas to the relevant information in each domain. Domains were further broken down into categories that were derivations of each domain by cross-analysis of the data (Hill et al., 2005). Based on these discussions and consensus reached by the team members, the category list was generated. This process took an entire academic year (October 2018 through June 2019). Finally, the domains and core ideas formulated during this phase were transferred to the chief researcher who served as an auditor and studied the data to identify the biases created in the group thinking process. Next, all the ideas that were formulated were grouped into categories and themes, and their frequency was counted. This process elicited a set of three main categories (Hospital teachers’ perception of their professional identity, Work context and Job satisfaction) and four sub-categories (*Hospital teaching goals, Teaching roles, Teaching skills, Working in a multi-dimensional staff)*.

According to Hill and colleagues (2005), if a category or a theme appears in the discourse of more than half of the participants (in our case over 18 participants), it should be termed ‘typical’ and identified as **most participants** having so responded in a certain manner. Themes cited by fewer than half the participants, but more than 10 participants were termed as ‘some’ and identified as having been responded to by **some participants** in a certain manner. Low-frequency topics (up to 10 participants) were associated with similar themes (Hill et al., 2005), and sometimes if important information was added, the findings were presented in the results section. In the final stage, all the qualitative materials were re-examined by the main author to determine their analysis in a comprehensive manner.

## Results

To gain understanding about hospital teachers’ unique professional identity, we asked teachers to reflect on their professional teaching environment (context) and roles, and how this influenced the construction of their professional identity. All data **was** analyzed and compiled into three categories and four subcategories. The first category addressed the direct answers to questions about how hospital teachers perceive their professional identity. The second category and sub-categories addressed the hospital teaching context, and the third category addressed the job satisfaction of hospital teachers.

### Category 1: Hospital teachers’ perception of their professional identity

Most hospital teachers in this study maintained that their professional identity is very difficult to define. They argued that the school setting and their roles are very different than those of the teacher in a community school thus influencing their professional identity in many ways. About half of the teachers claimed that their professional identity stems from their main role which is to provide the student **continuity of education**. They believe that as teachers they are “child experts” and therefore, even when a child is a way from school, he/she is entitled to continue and engage in an adjusted educational dialogue focused on promoting normal self-development. Several teachers said that their main professional identity is to be regular teachers and **promote normalcy** in the medical center by maintaining a regular school environment and teaching standard academic contents.

“I really find it very difficult to define my professional identity …. I am a teacher or maybe an educator …I provide education, but in a totally different matter… (Teacher, 11, Child Cardiology department).

“I think teachers are part of the child’s normal development….and when he/she is away from school, they still need to feel like normal kids” (Teacher, 2, Child Oncology department).

### Category 2: Teaching Environment (context)

All hospital teachers in this study described the teaching environment as unique, beginning with the fact that hospitalized children are there to receive necessary medical treatments and learning is only secondary to the medical processes. In addition, children may be hospitalized due to a wide variety of medical conditions and often suffer from physical pain and negative emotions; some are in acute crisis. Moreover, children are admitted to hospital for different periods of time, from several hours up to several months. Teachers claim they can never know on any given day who their students will be; their age, medical condition, mother tongue, as well as, what personal developmental, and learning needs they will have. Teachers say they must be responsive adapting quickly to the unknown, changing environment and varying conditions.

“I arrive in the morning, do the rounds between the departments, ask the medical staff if there are any special needs – for example, children who go to all kinds of procedures or tests, see who is new and who has arrived, and invite them to our classroom. You have no idea who will come and therefore you need to be very attentive and creative” (Teacher, 22, child Orthopedics department).

“I do not have a regular, structured classroom. I approach children who are the more difficult cases, those who refuse treatment, are suicidal, etc. The staff in the department know that those kids are close to my heart, and they contact me and connect me with the children.”(Teacher, 15 child Psychiatry department).

#### Sub-category 2a: Hospital-Teaching Goals

When teachers were asked about their educational goals, there seemed to be a great deal of confusion and the responses were very general. Most teachers claimed to be “part of the sick child’s healthy world”, maintaining that it was their job to talk to the child about his normal life. They said their primary goal was to support the child’s recovery process through educational means such as academic learning and expressive tools. Several teachers said the goal of their work was to help the hospitalized child develop strengths with which to better cope with the hospitalization, the illness, and its possible future implications. Few replied that the goal was to complete educational deficiencies, teach learning skills, and maintain contact with the enrolled school.

“Sometimes you teach, but most of the time the children come to hospital for a few days and do not really lose educational material …. And they do not want to learn …. So, we try to be there and make it easier through games and discourse” (Teacher 30, child Dialysis department).

“The kids know teachers from their daily routine, they know what schooling is and so they just relax and cooperate with us because we are part of their healthy world” (Teacher 8, child Surgery room).

Most of the teachers noted that the learning processes and the learning content in the hospital are very different from those in the enrolled schools. They noted that in the hospital there is no real importance to the knowledge taught in the enrolled school, since the student is not in a normal state of knowledge absorption and usually remains inhospital for only a short period of time. Most teachers stated that they try to make the subject matter relevant to the child’s general medical condition, social or cultural events (e.g., holidays), or current affairs.

“I am a math teacher and love teaching that subject, but often when a child comes and I ask him if he wants to study with me, he will ‘make a face’ and sometimes even openly oppose …. I do not want to impose irrelevant learning and therefore try to teach them something relevant to their medical situation” (Teacher, 2, child Oncology department).

“It is very difficult to decide what to teach, especially if the student does not speak Hebrew…. and there is no Arabic-speaking teacher currently in the class …. but I do not give up and try to play some didactic game” (Teacher 1, child Dialysis department).

#### Sub-category 2b: Hospital Teaching Roles and Tasks

Most of the teachers described varied roles and tasks in which they engage. They described individual and group lessons, preparation for matriculation exams, artistic lessons, and relevant medical oriented lessons in anatomy, physiology, also including preparation for medical procedures. The teachers reported teaching about current events, social and cultural matters, as well as, lessons in self-management, meditation, and mindfulness. They spoke of playing with children, calming parents with their children, and mediating the hospital environment to them. More rarely (this is true mainly for teachers in the oncology departments), teachers visit the enrolled school of the child and meet with teachers and friends to explain the condition of the hospitalized child.

“I teach high-school students in the psychiatry department, preparing them for matriculation exams, and most of the time I’m busy with math and English … the main thing is that they go to the exam feeling prepared, and that they have not accumulated a lot of gaps.” (Teacher, 2, child Oncology department).

“Nobody in the department really understands what my exact role is here and it’s hard for me to explain and demarcate boundaries.” (Teacher 13, child Endocrinology department).

“In the oncology department there is a lot of work with parents. They are under great pressure, sometimes they fail to understand what the doctor is saying and are very much in need of reassurance … because the medical team is not there every day, all day like we are we do lots of supportive work with parents” (Teacher 7, child Oncology department).

#### Sub-category 2c: Hospital Teaching professional Skills

When the teachers were asked what guided them in their educational work at the hospital, the vast majority spoke of their own basic intuition and a great deal of creativity. They all agreed that without the quick ability to respond and improvise, they would never have survived in this job because after all, they had never been trained and the boundaries of the job were very unclear. Sometimes the guidelines for action come from the medical team, the paramedics, and other times from the educational management but, mostly the teacher creates her own framework in the department according to the needs of the moment. The teachers reported that very often they sense that what they do overlaps the work of the art therapist, the social workers and even the nurses, and because they are all present in the department every day, they are more readily available. At the same time, the hospital teachers describe their status as less significant relative to the other professionals.

“For a moment I am a teacher, then I become a therapist … an actor …. I need to be very sensitive, flexible, and able to improvise …. I need to be emotionally competent to survive, but also enjoy my job…. I’m there so the student will feel good.” (Teacher 24, child Endocrinology department).

“Sometimes I feel like a kindergarten teacher, and I play with students or paint with them, tell a story and sing….. sometimes I prepare children for a medical procedure ….and sometimes teach Math. I feel I need to be here very empathic, creative and sensitive to everyone’s needs at a given moment… nobody really teaches you the skills for being a hospital teacher” (Teacher 18, child Cardiology department).

#### Sub-category 2d: Working in a multi-dimensional staff

Hospital teachers report that they work with a multi-professional team. Their “boss” is a medical doctor, and their colleagues are mostly the medical staff. They find it interesting but complex to work in such a team, where nobody really understands the educational aspects and goals and often confuse it with occupational therapy or babysitting. Most teachers reported feeling undervalued by the medical staff.

“Without a doubt, we perform a lot of functions here in the department but when there are staff meetings we are not always invited, and they are not adamant about hearing what we have to say ……” (Teacher 30, child Dialysis department).

“In the regular school in my previous job the teacher is still half God ……. we decide … we make the most significant decisions during the day. Here we have little freedom and are always subject to the medical routine.” (Teacher 37, child Orthopedics department).

“Earlier, when a doctor would make a remark to me or rush the preparation for a procedure, I was silenced and thought that the main thing would be the medical process. Today I understand better and tell the doctors that the child needs the preparation time, and that the child’s emotional world is no less important than his physical condition” (Teacher 8, child Surgery room).

### Category 3: Hospital teachers’ job satisfaction

Most of the teachers interviewed for this study claimed to be very satisfied with their work but noted that it is also draining and very challenging. They emphasized the need for unique training and support, as well as, the issue of cumulative emotional difficulty, diffused professional identity, and a need for recognition. However, all these points were secondary to their consistent feelings that they had been awarded significant, interesting, and unusual educational work that they would not exchange for any other work. Most of the teachers remarked that they wanted to see the field develop and become a real profession, and some were willing to contribute from their knowledge and experience.

“Sometimes during the work day, I don’t even feel like I’m at work …… I don’t have to stand up in front of a classroom, find the time to teach irrelevant study material…. here I have fun with the kids …. I feel happy and satisfied.” (Teacher 16, child Endocrinology department).

“Being with these children is satisfying, enriching and usually very exciting …. The difficulty stems mainly from the confusion and the sense that you do not quite understand what your unique role is”. (Teacher 15, child Psychiatry department).

I feel it is challenging to work with ill children that are in pain, or very anxious about their medical situation … Some of them are confused, upset, angry …. It is also difficult to work with an adolescent when a parent is present most of the time…. But I feel that this work is very meaningful, and I can make a significant contribution to their hospitalization experience…(Teacher 30, child Dialysis department).

### Summary

The results section addressed different aspects in hospital teachers’ perceptions of their professional identity. The first category revealed the uniqueness of this professional identity due to the different educational context and goals. The second category and its subcategories elaborated on the hospital teaching context, roles and tasks indicating the characteristics of this job, its challenges, and complications. The third category explored hospital teachers’ job satisfaction. All together these results suggest a unique, challenging and satisfying professional identity.

## Discussion

Teachers in hospitals work in a unique educational setting (Steinke et al., 2016). They face goals, tasks, and instructional environments that require creative and flexible educational practices that differ from those which they were originally trained for, and those practiced in the base schools (Csinady, 2015). Following Akkerman and Meijer, (2011) it was assumed that since hospital teachers work in a unique educational environment this job context would influence their perceived professional identity in many ways (Capruso & Dennis, 2017; Henry, 2016). Understanding the importance of teachers’ professional identity to the practice of their craft and their job satisfaction (Canrinus et al., 2011), the present study examined hospital teachers’ professional identity from their own perspective and experience. We assumed that a more accurate understanding of one’s own professional identity would contribute to the quality of hospital teacher’s professional life and practice, as well as, to the overall understanding and promotion of the profession (Akkerman & Meijer, 2011; Hen, 2018).

Our findings revealed that hospital teachers define their professional identity as focusing both on *continuity of education* and *promoting normalcy* in the medical milieu. When trying to understand how these focuses transfer into hospital teachers’ professional identities, varied educational practices, different ways and means of teaching, and the complexity of working with hospitalized children, parents, and a multi-professional team were presented. However, when directly asked about their professional identity, teachers found it difficult and confusing to define their professional identity and educational goals. Finally, hospital teachers reported being very significantly satisfied with the educational work in the hospital and expressed the desire to learn more and develop themselves in this specific profession.

These findings align with the literature that suggests teachers’ professional identity is an elusive concept that refers to a dynamic match between an individual’s internal processes and actual external scenarios (Henry, 2016). It seems that the fact that hospital teachers work in an educational setting that differs tremendously from the common school setting (Capruso & Dennis, 2017) affects their inner professional dialogue and challenges them to reconstruct the traditional teaching roles and professional identity to fit this unique educational environment (Benigno & Fante, 2020). For example, redefining the meaning of teaching for the transfer of general knowledge to teaching specific medical oriented knowledge to help hospitalized student reduce anxiety (Hen, 2020).

Also, the traditional social expectations from hospital teachers often do not align with the educational roles of hospital teachers, and challenge the interaction between the self, the socially expected and the work context (Äärelä et al., 2016; Flores & Day, 2006). For instance, hospital teachers feel that their educational roles are often unclear to parents and to the medical staff, and they are asked to perform all kinds of tasks that interfere with their educational work (Hen, 2018; Steinke et al., 2016).

Interestingly, a plenary of literature on teachers’ professional identity focuses on the discourse occurring in a person during the training process, and its significance for future formulation of the professional identity (Cohen, 2010; Izadinia, 2015). During that process, the teacher negotiates the different aspects of the profession and understand its demands, while examining how these relate to their own attitudes, values, and personality (Beijaard et al., 2004). However, hospital teachers do not receive training dedicated specifically toward their role as hospital teachers and arrive at the job armed only with their previous training as a teacher in a conventional school setting (Beningo et al., 2018). Thus, they need to negotiate and consolidate their unique identities as hospital teachers ‘on the job’, without the luxury of time or considerations they may have had in other circumstances (Hen, 2020; Cohen 2010; Izadinia, 2013, 2015). In addition, it must be remembered that in the hospital setting, teachers encounter complex professional dilemmas they had not seen previously (e.g. an inpatient child is not obliged to study when he is hospitalized), they deal with a complex and multifaceted work environment (working with a multi-professional team) and they are faced with very dynamic and changing situations that require a great deal of flexibility (Hopkins, 2016).

This state of diffused professional identity, which is persistently challenged, is not necessarily a bad professional situation, but the literature does encourage a substantial and continuous discourse to help teachers recognize and connect their various personal and professional entities (Rodgers & Scott, 2008). This cohesion of different personality elements allows teachers to simultaneously feel their professional identity is firm and clear yet flexible, and allows for creativity, initiative, and plus diversity in their professional conduct at the hospital (Akkerman, & Meijer, 2011; Csinády, 2015). According to the literature, teachers in hospitals like other teachers need to develop a clear sense of their role as teachers together with their ability and commitment while still maintaining an attentive and open professional identity that will meet the needs of the actual situation (Benigno & Fante, 2020; Canrinus et al., 2012). Teachers must provide a professional educational solution for the hospitalized child and his/her parents, while at the same time working fully and mutually with the members of the medical and paramedical staff (Eaton, 2012; Steinke et al., 2016).

All these undoubtedly challenge the professional identity of the hospital teacher and raise the need for professional discourse to support the decisions they make and the way in which they work with children, parents, and medical staff (Hen, 2020; Benigno & Fante, 2020). It should be considered that the teacher’s professional identity also relates to their motivation to advance and develop, to their professional quality of life and to the prevention of burnout (Canrinus et al., 2012) therefore, the importance of discourse on this topic is far beyond the practical aspect.

This may suggest that further emphasis should be given to how the interaction between the work context and hospital teachers’ inner voice construct their professional identity and to further create the opportunities for hospital teachers to engage in professional dialogues. This may allow them to develop a clear professional identity that is simultaneously unitary but also multiple, continuous, and discontinuous, and individual but, also social (Akkerman & Meijer, 2011; Benigno & Fante, 2020; Cohen 2010). Through professional development opportunities hospital teachers will be able to better define their specific and unique roles, feel more confidence in their professional position and better distinguish it from other professions in the multidimensional staff (Hen, 2020; Benigno & Fante, 2020).

Finally, in the present study, most of the teachers reported a high degree of satisfaction in their work, despite the ambiguity surrounding their professional identity. However, a thorough examination of the data shows that satisfaction is due mainly to the personal interest and emotional rewards that exist in this work and not necessarily to professional aspects such as teaching and maintaining the educational continuum (Beningo et al., 2020; Csinady, 2015).

### Limitations

Despite the interesting findings of this study, one should keep in mind that this study was a qualitative, preliminary study based on semi-structured interviews and a sample of staff members in only three medical centers in one country. To reinforce the findings of the study, it is highly advisable to repeat it in other settings, and to use additional research methods to expand understanding of hospital teachers’ professional identity. In addition, our sample was relatively small and included only female teachers.

## Conclusions and Implications

This study explored the professional identity of hospital teachers as reflected in their own experience and voice. The findings strengthened the understanding of the unique and complex educational environment where hospital teachers work and the importance of developing a clear professional identity. The central conclusion of this study is that hospital teachers need unique and specified training that will allow them to formulate their professional identity, along with a continuous professional discourse that will help them connect the professional and personal parts into a clear and flexible professional identity.

Research in this area would appear to improve the quality of life of teachers in hospitals and their professional work by promoting its general understanding and subsequent development. It is also important to examine the work of hospital teachers through the eyes of other involved groups, such as the medical and paramedical staff, the parents, and the children themselves. In future studies, it may be useful to examine additional variables such as working with a targeted population of hospitalized children (different ages, different illnesses, different cultures), with defined educational tools (interactive board, computers, demonstration dolls), and investigating the personal well-being of hospitalized children.
